# Metagenomic analysis and biodiversity of bacteria in traditional fermented fish or Budu from West Sumatera, Indonesia

**DOI:** 10.5455/javar.2023.j736

**Published:** 2023-12-31

**Authors:** Yetti Marlida, Malikil Kudus Susalam, Harnentis Harnentis, Jamsari Jamsari, Nurul Huda, Wan Norhana Md Noordin, Lili Anggraini, Laily Rinda Ardani

**Affiliations:** 1 Department of Animal Nutrition and Feed Technology, Faculty of Animal Science, Andalas University, Padang, Indonesia; 2 Graduate Program, Faculty of Animal Science, Andalas University, Padang, Indonesia; 3 Department of Genomic and Molecular Breeding, Faculty of Agriculture, Andalas University, Padang, Indonesia; 4 Faculty Sustainable Agriculture, Universiti Malaysia Sabah, Sandakan, Malaysia; 5 Fisheries Research Institute, Batu Maung, Malaysia

**Keywords:** DNA sequencing, fermented fish, metagenomic, Sumatra Budu

## Abstract

**Objective:**

This research aims to investigate the microbial diversity of Budu prepared from fresh and frozen fish from the Pariaman and Pasaman districts in West Sumatra Province, Indonesia, as well as provide basic information about Budu quality.

**Materials and methods:**

To obtain the bacterial microbial composition, deoxyribonucleic acid extraction was carried out using amplicon-sequencing of the *16S-rRNA* gene in the V3–V4 region from two types of Budu and carried out in duplicate.

**Results:**

Budu prepared with fresh (Pariaman) or frozen (Pasaman) fish was dominated by Firmicutes (78.455%–92.37%) and Proteobacteria (6.477%–7.23%) phyla. The total microbial species in Budu from Pariaman were higher (227 species) than in Pasaman (153 species). The bacterial species found are *Lentibacillus kimchi* (1.878%–2.21%), *Staphylococcus cohnii* (0.597%–0.70%), *Peptostreptococcus russeli* (0.00%–0.002%), *Clostridium disporicum* (0.073%–0.09%), *Clostridium novyi* (0.00%–0.01%), *Nioella sediminis* (0.00%–0.001%), and *Shewanella baltica* (0.00%–0.003%). *Lentibacillus kimchi, S. cohnii,* and *C. disporicum* are found in both Budu. *Nioella sediminis* and *S. baltica* are found in Budu Pariaman. *Peptostreptococcus russeli* and *C. novyi* were found in Budu Pasaman.

**Conclusion:**

Metagenomic analysis of Budu from different fish, Pariaman (fresh fish) and Pasaman (frozen fish) showed that the biodiversity of bacteria was barely different. Both Budu found lactic acid bacteria from the *Enterococcaceae* family, genus *Vagococcus,* and pathogenic bacteria, such as *S. cohnii, P. russeli, C. disporicum,* and *S. baltica*. The discovery of various species of pathogenic bacteria indicates that development is still needed in the Budu production process to improve Budu quality.

## Introduction

Fermentation is the most economical method of food preservation and has been used by our ancestors for a long time. Fermentation extends shelf life, reduces volume, shortens cooking time, enriches the taste and aroma, and increases the bioavailability of the product. Fermented foods serve as functional foods that are beneficial to health. West Sumatra province in Indonesia is famous for its traditional fermented fish, locally known as Budu. Budu is usually prepared from marine fish such as queenfish (local name: Talang, *Chorinemus* spp.) or mackerel (local name: Tenggiri, *Scomberomorus* spp.) by coastal communities and marketed locally [1]. Budu is regarded as a delicacy, a homemade artisanal product with minimal mechanization or technology. It is produced through a long natural and spontaneous fermentation involving mixed beneficial microbes from the fish itself and surrounding environments to ensure the solubility of the fish mixture.

In general, Budu preparation begins with hanging the fresh or frozen fish with the head down for 12 h. The digestive tracts were then removed, and the fish was soaked in salt water (at a concentration of 20% of the fish’s weight) for 3 days. The fish were washed thoroughly, dried for 7 days under the sun, and fermented aerobically in a box at ambient temperature (25°C–30°C). The final product can have a different composition at different times and places due to the fermentation that is carried out naturally and spontaneously by the producer [1–3]. The process of making fermented food traditionally utilizes natural microbes found in the raw materials that make it [4].

Understanding the composition of the microbiota population is important to further improve the quality of Budu. Currently, generally, to profile the microbiota of culturable and non-culturable fermented foods, methods that do not depend on culture can be used using deoxyribonucleic acid (DNA) samples from fermented foods [5–8]. Previous reports documenting complete profiles of microorganisms revealed inter- and intraspecies diversity within a genus or between specific genera. Currently, next-generation sequencing (NGS) is a tool that can be used for molecular identification, such as metagenomics, phylogenetics, and transcriptomics, which are currently applied to the cultural documentation of various samples. This method is well accepted as an accurate and reliable identification tool that can be used in the identification of microbial communities present in kimchi from Korea [9,10], Godo, fermented soy from Japan [11], Lemea, fermented bamboo shoots and fish from Indonesia [12], Incwancwa, a traditional fermented cereal from Eswatini [13], and Masin (salted sauce) in Sumbawa, West Nusa Tenggara, Indonesia [14].

Until now, only a few reports have reported on the diversity of Budu microbiota from Indonesia, especially through molecular approaches. Exploration of the diversity of microbes found in fermented products, especially food, can be an indicator of the quality of fermented food, especially fermented fish. Thus, this research was conducted to explore the diversity of Budu microbes using metagenomic technology to obtain information to improve the quality and consistency of existing local culinary delights. Through this research, the diversity of potential bacteria and pathogens in Budu will be discovered, providing basic information for Budu producers to develop Budu products and increase their added value.

## Materials and Methods

### Ethical approvals

This study did not use any live animals, so it does not require ethical approval.

### Source of Budu

**Figure 1.**
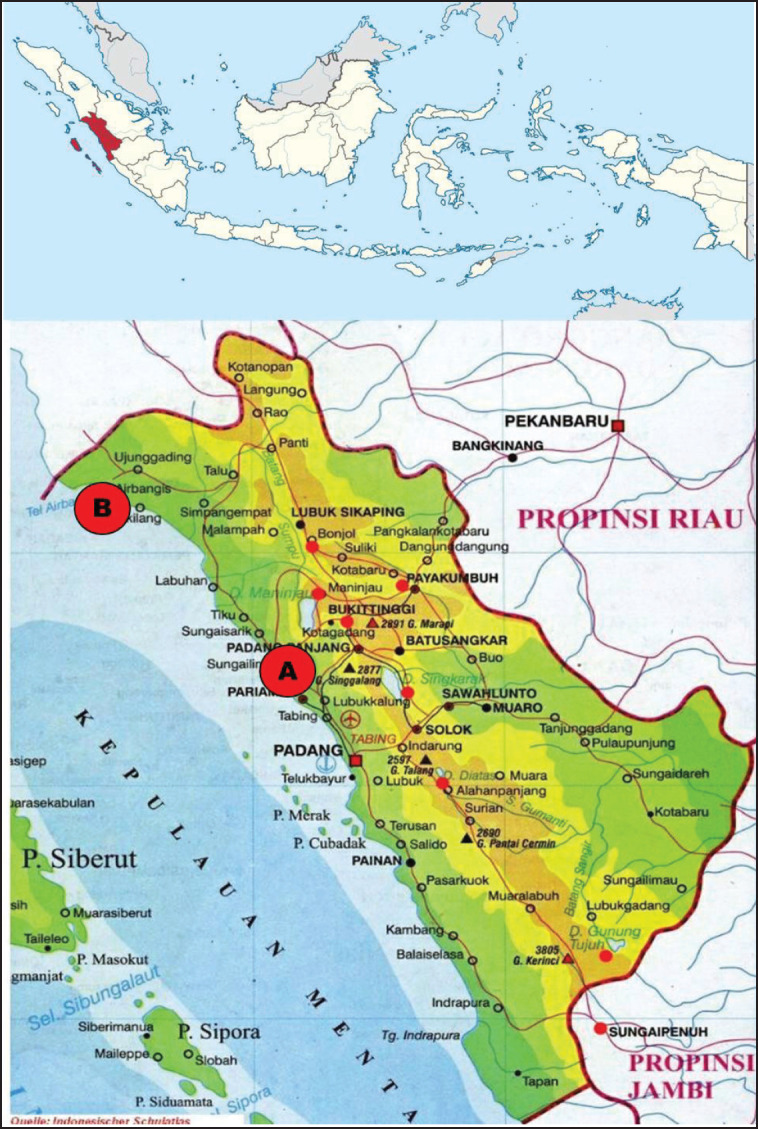
Two locations of budu collection from West Sumatera Province. A: Pariaman and B: Pasaman.

The Budu used in this research came from two types of locally produced Budu collected from the Sungai Limau area, Padang Pariaman Regency, and Air Bangis, Pasaman Regency, West Sumatra Province, Indonesia. The Budu from the Sungai Limau area were made from fresh fish [Mackerel (*Scomberomorus gutattus*)] (Fig. 1), whereas the Air Bangis Budu were prepared from frozen fish [Queenfish (*Chorinemus tala cuvier*)]. The fractionation of the samples to obtain DNA extracts, polymerase chain reaction (PCR) amplification, and DNA sequencing were carried out at the Laboratory of Genetika Science, Tangerang City, Indonesia.

### Extraction of DNA

The DNA extraction process from Budu samples was carried out by centrifuging at a speed of 14,500 g (Centurion K243R, Netherlands) to obtain pellets. Next, the DNA extraction process was carried out using the Quick-DNA™ Microbe Kit from Zymo Research, USA (according to the manufacturer’s protocol). The homogenized tissue from the extraction process was lysed using a Bertin instrument in France.

### PCR amplification and NGS

PCR amplification was carried out in two steps to obtain amplicons from the 16S rRNA gene with the V3–V4 region. This study used forward primer 341F (5'-CCT ACG GGN GGC WGC AG-3') and reserve primer 785R (5'-GAC TAC HVG GGT ATC TAA TCC-3') from the Illumina kit (Illumina, USA). To analyze the resulting fragments, a technology from Advanced Analytical Technologies, Germany, and fluorometric analysis were used to purify PCR products. Next, the purified PCR products were examined using a fragment analyzer and quantified, multiplexed, clustered, and sequenced using Illumina MiSeq. The 16S rRNA sequence analysis was done using BaseClear (Leiden, The Netherlands).

### Sequence processing and analysis

The sequencing process used the Illumina CASAVA pipeline (version 1.8.3). FASTQC software (version 0.10.0) was used to perform raw sequencing, filtration, and trimming processes to obtain good data. Quantitative Insights Into Microbial Ecology (QIIME) software (version 1.9.1) was used for the subsequent analysis process [15] to obtain *α* and *β* diversity. To find biomarkers between groups using relative abundance from the operational taxonomic unit (OTU) table generated in QIIME, linear discriminant analysis (LEfSe) was used [16]. Finally, statistical analysis of the multivariate data using METAGENassist [17].

**Figure 2.**
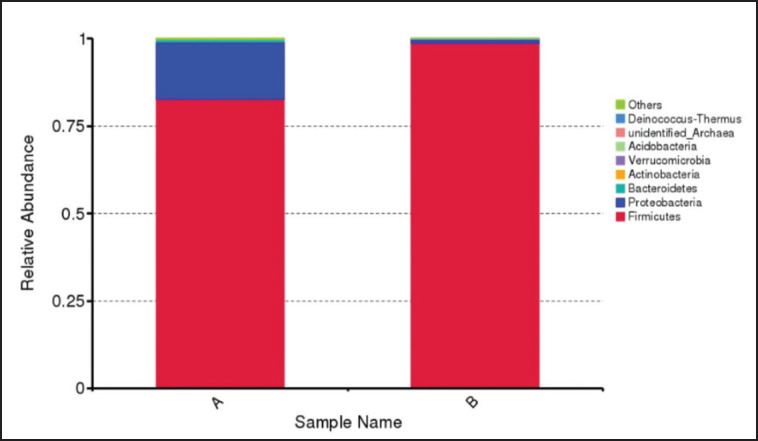
Relative abundance of microbial community in budu. Composition of microbial community based on metagenomic analysis. A: budu from Pariaman, B: budu from Pasaman.

**Table 1.** Number of microbial species of from two types of budu (A: Pariaman and B: Pasaman) based on DNA sequencing in the V3–V4 region of the 16S rRNA.

Sources of budu	No of microbial species	Shannon	Simpson	Chao1	ACE	Goods-coverage	PD-whole_tree
A (Pariaman)	227	1.552	0.371	234.241	236.815	1.000	31.244
B (Pasaman)	153	2.209	0.611	164.757	176.327	1.000	16.400

## Results

### Composition of the microbiota

**Figures** represent the overall diversity of the microbial community and the relative abundance of microbiota recorded in Budu samples from two different locations in West Sumatra. Analysis of the two Budu found several types of bacteria from the phylum *Firmicutes, Proteobacteria, Bacteroidetes, Actinobacteria, Verrucomicrobia, Acidobacteria,* and* Deinococcus-Thermus* (Fig. 2).

**Figure 3.**
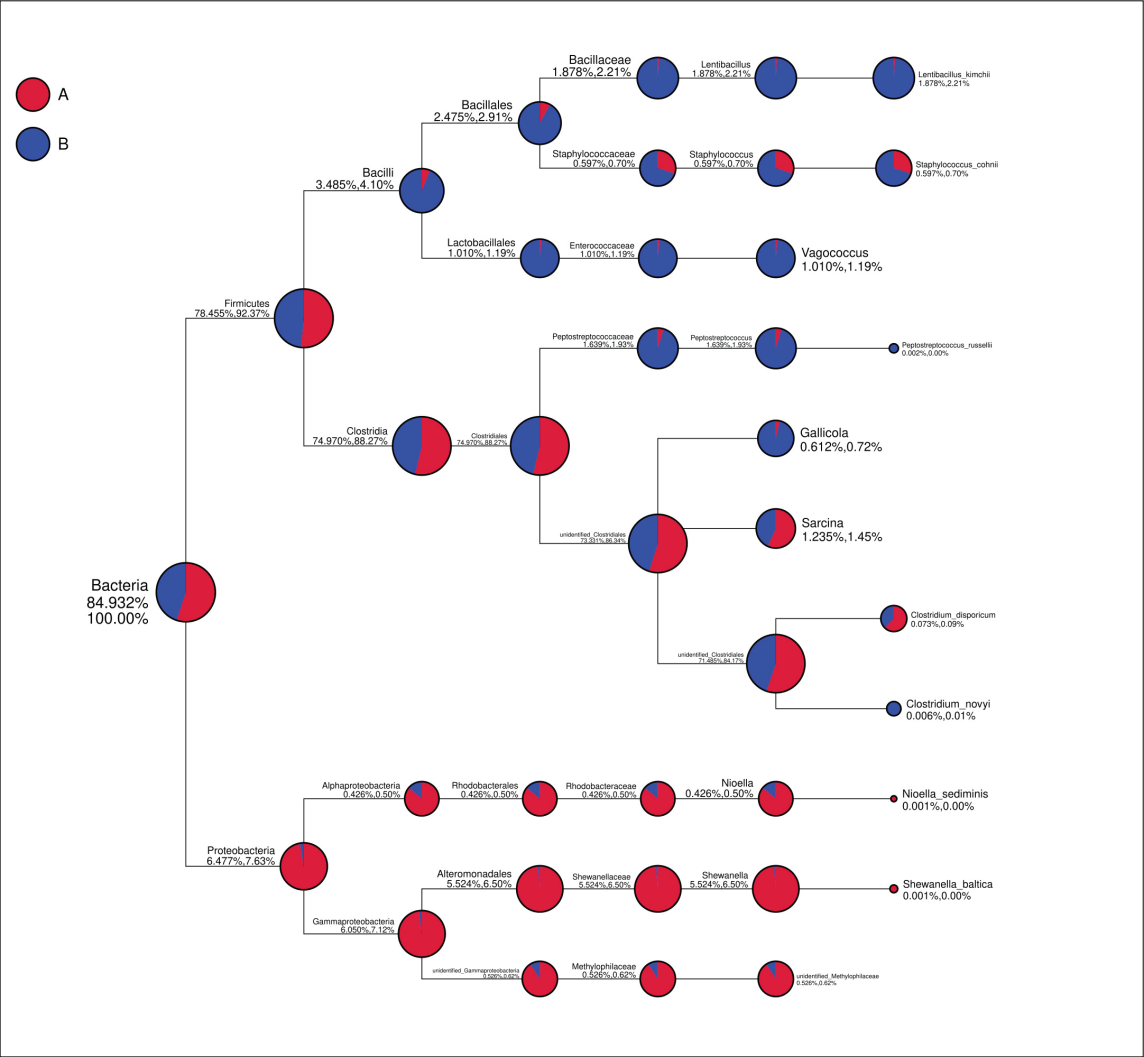
Taxonomy tree of budu from A. Pariaman and B. Pasaman. Taxonomy of bacteria in budu based on metagenomic analysis. Overall diversity and relative abundance of 16S microbiota of the budu from Pariaman (made using fresh fish) and Pasaman (made using frozen fish).

**Figure 4.**
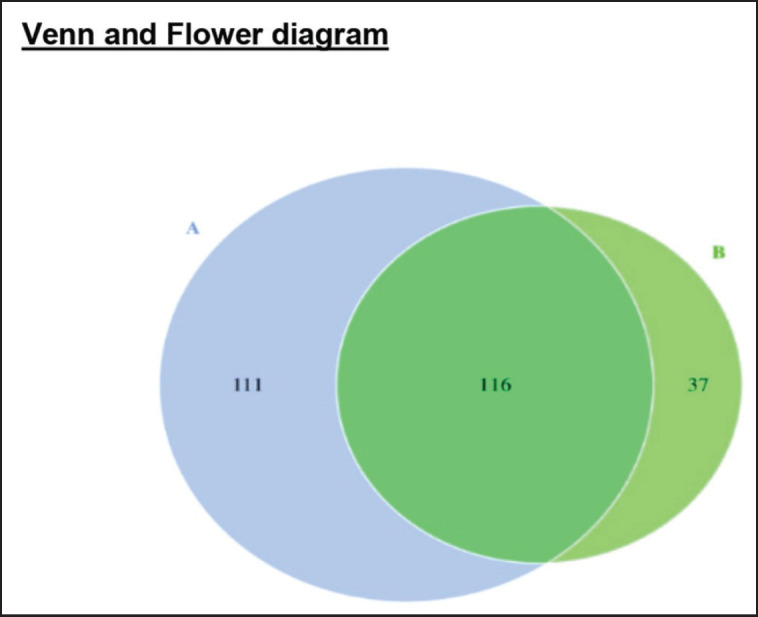
Venn diagram of the distribution of bacterial OTUs in budu samples from two different areas. A: Pariaman and B: Pasaman.

There was only a slight variation in the microbiota composition of Budu from Pariaman to Pasaman. The results of the microbiota in Budu products from Pariaman and Pasaman showed that it was dominated by the Firmicutes phylum, with a relative abundance reaching 78.45%–92.37% (Fig. 3). Meanwhile, the Proteobacteria group as the second most abundant microbiota was shown, with a relative abundance reaching 7.63% (Fig. 3). In the *Firmicute* phylum, bacteria from the *Clostridia* class (74.97%–88.27%) and* Bacilli* (3.485%–4.1%) were detected, while in the *Proteobacteria* phylum, bacteria were found in the *Gammaproteobacteria* class (6.05%–7.12%) and *Alphaproteobacteri* (0.426%–0.50%). The number of total bacterial species in each Budu sample was also distinguished (Table 1). The total microbial species in Budu from Pariaman were higher (227 species) than in Budu from Pasaman (153 species). Venn diagrams are used to display common and unique OTUs showing two regions with different raw materials and processing methods for making Budu. From the total of 227 species detected in the Budu sample from Pariaman, 116 of them were also noted in Budu samples from Pasaman (Fig. 4). Only 111 microbial species were exclusively noted in Pariaman and 37 in Pasaman Budu samples (Fig. 4).

### Phylogenetic microbiota in Budu

**Figure 5.**
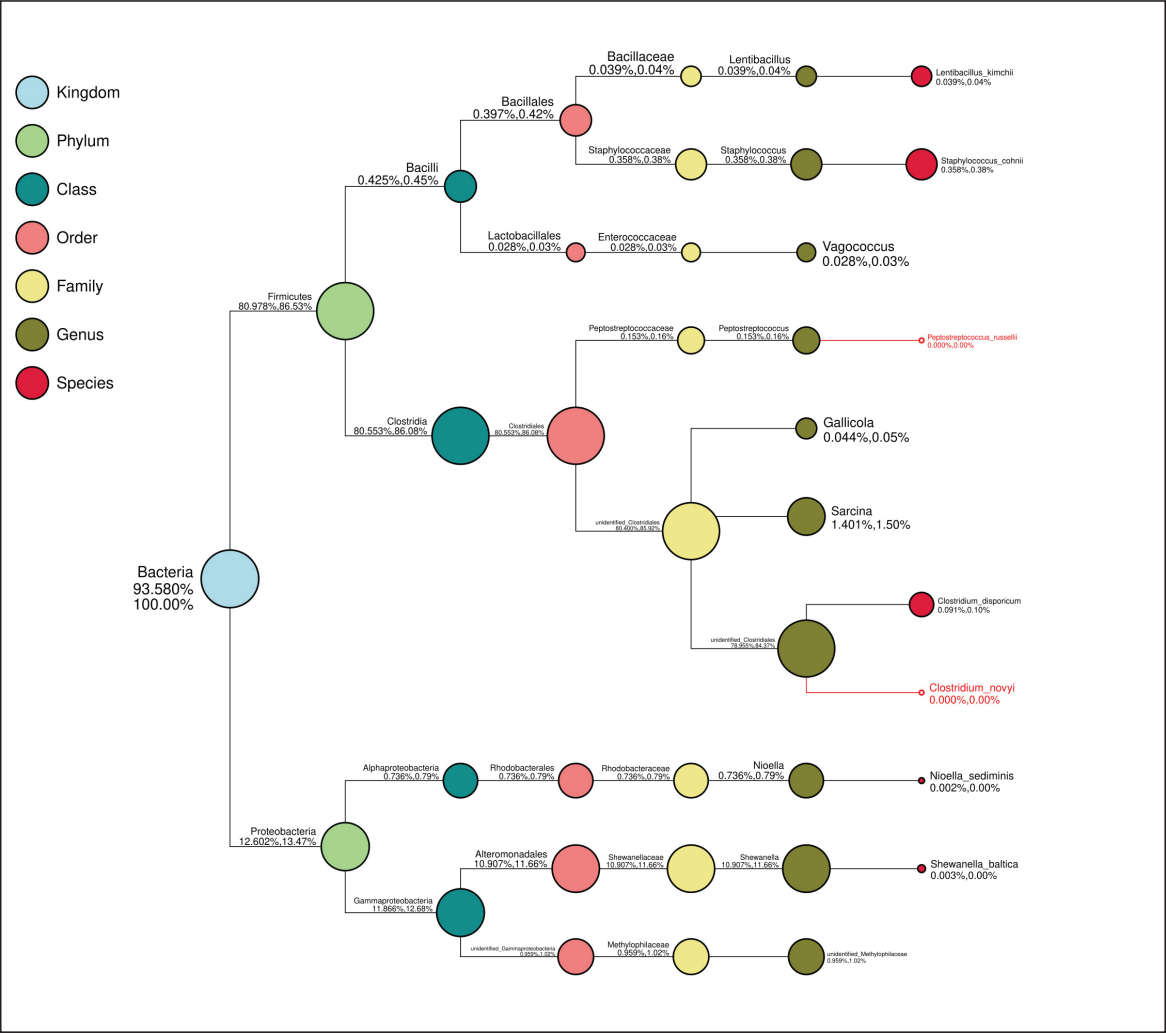
Phylogenetic tree of bacteria found in budu based on metagenomic analysis. Larger circles/signs indicate the greater number of bacteria at each taxon level which is also indicated by the percentage.

Figure 5 illustrates the detailed microbiota analysis in Budu from Pariaman and Pasaman. Bacterial was identified as the main microbial kingdom in both Budu products, with a percentage of 93.58%–100%. Two phyla of bacteria and several species were found in Budu samples. Budu were dominated by the *Firmicutes* phylum with a percentage of 80.978%–86.53%, followed by *Proteobacteria* with only 12.602%–13.47%.

Clostridia is the main bacterial class under the *Firmicutes* phylum, with a percentage of 80.551%–80.08%. The genus identified under the *Clostridia* class are *Peptostreptococcus* (0.153%–0.16%), *Gallicola* (0.044%–0.05%), *Sarcina* (1.401%–1.50%), and presence of an *unidentified Clostridiales* genus (78.955%–84.37%). At the species level, it was identified as the presence of *Clostridium disparicum* (0.091%–0.10%).

Bacteria belonging to the *Bacilli* class under the *Firmicutes* phylum have a presence percentage of 0.425%–0.45%. The genera identified under the *Bacilli* class are *Lentibacillus* (0.039%–0.04%), *Staphylococcus* (0.358%–0.38%), and *Vagococcus* (0.028%–0.03%). *Lentibacillus kimchii* (0.039%–0.04%) and *Staphylococcus cohnii* (0.358%–0.38%) are species identified as belonging to the *Bacilli* class.

On the other hand, two main classes of bacteria dominating the *Proteobacteria* phylum were particularly *Alphaprotobacteria* (0.736%–0.79%) and *Gammaproteobacteria* (11.86%–12.68%). The main genus under *Alphaprotobacteria* was *Nioella* (0.736%–0.79%), while two main genera dominated the Gammaproteobacteria class, i.e., Shewanella (0.003%) and *unidentified Methylophilacese* (0.959%–1.02%). The species detected were *Nioella sediminis* (0.02%) and *Shewanella baltica* (0.003%).

## Discussion

There was only a slight variation in the microbiota composition of Budu from Pariaman to Pasaman. Analysis of bacterial metagenomic data from two Budu samples only found two phyla, *Firmicute* and *Proteobacteria* (Fig. 2). Microbiota diversification in fermented fish products (Pla-ra) from three different places in Northeast Thailand was previously reported on the identification of 17 bacterial phyla divided into many families and genera [18]. Meanwhile, bacterial strains of 11 phyla and 29 genera were observed in fish sauce in China [19]. Zoqratt and Gan [20] reported the five most abundant phyla in Budu samples from Malaysia, comprised of *Firmicutes* (60.26%), *Proteobacteria* (22.86%),* Halanaerobiaeota* (10.60%),* Actinobacteria* (3.91%), and *Bacteroidetes* (1.91%). Identification carried out on Rakfisk (traditional fermented fish brine) from Norway taken from six producers during two years of production succeeded in finding 65 genera of bacteria and one genus of archaeal [21].

We observed that from this study, the total microbial species (Table 1) from Budu in Pariaman (227 species) were higher than those from Budu in Pasaman (153 species). This is thought to be caused by differences in the types of fish raw materials used for research. Logically, the fresh fish used in Pariaman will have more bacterial species compared to the frozen fish used in Pasaman. The fermentation process and duration, raw materials used, salt concentration, and recipe cause different microorganism results between samples [18].

In addition, other studies report that the presence of variations in the types of microbes in fermented foods results in differences in quality, nutritional composition, and resulting health benefits [22,23]. *Firmicutes* and *Proteobacteri*a were also the main genera in Budu from Malaysia [20]. Firmicutes is a genus of bacteria included in gram-positive bacteria. Megasphaera,* Pectinatus*,* Selenomonas*, and *Haemophilus* have a porous pseudo-outer membrane (lipopolysaccharides), which causes them to be gram-negative in color.

The bacterial species detected in Budu from Pariaman and Pasaman, West Sumatera, were different from the species reported in other fermented fish products. Previous research has found a broad spectrum of microorganisms in fermented fish products in Asia [24], since different types of raw materials and fermentation conditions produce different microbiota. Meanwhile, when compared with masin products (fermented sauce made from shrimp paste, chilies, turmeric flowers, and other spices originating from Sumbawa, West Nusa Tenggara, Indonesia), the class of bacteria most frequently detected was the *Enteroccocaceae* family, genus *Tetragenoccocus* [14,25]. Previous studies reported that bacterial and yeast microbiota originating from Budu were able to increase feed digestibility and act as agents in inhibiting aflatoxins B1 [26,27].

In our explorations, as many as six species of bacteria were found in this fermented product, including those from the genera *Bacillus subtilis*,* Staphylococcus arlettae*,* Enterobacter cloacae*,* Leuconostoc mesenteroides*,* Staphylococcus petrasii*, and *Escherichia coli.* All of them were recorded in traditional fish fermentation (Bekasang Loar) in Indonesia [28]. In another report, a total of 134 strains came from Budu fish from Malaysia, with the highest yield of *Micrococcus* sp. (32.1%), followed by *Staphylococcus* (27.6%), *Pediococcus* (10.4%), and *Candida* (8.9%) [29]. Gammaproteobacteria and Bacilli dominated the microbiota of Rakfisk [21]. *Tetragenococus halophilus* is a species whose presence dominates in fermented fish sauce originating from Vietnam [30], and *Lactococcus* and* Lactobacillus* were also abundantly isolated from Yucha and *Vibrio. Acinetobacter* and *Enterococus* were also detected with a relatively low abundance [31]. A similar observation was noted in Hongeo (South Korea), where the different bacterial populations were associated with different fermentation environments, including temperature, humidity, and others [15].

Metagenomic analysis showed that in the Budu fish samples, there were several groups of lactic acid bacteria (LAB) from the *Enterococcaceae* family, *Vagococcus* genus. LAB produces various organic acids as end products of carbohydrate fermentation, which include acetic acid, succinic acid, propionic acid, and lactic acid. The organic acids produced by LAB can prevent the growth of pathogenic microorganisms by lowering intracellular pH and inhibiting the active internal transport of excess protons, which involves cellular consumption of adenosine triphosphate and depletes cells. Organic acids attack the bacterial cell wall, cytoplasmic membrane, and bacterial-specific metabolism, leading to the destruction and death of pathogenic microorganisms [32].

Besides LAB, there are also pathogenic bacteria in Budu, such as *S. cohnii, Peptostreptococcus russeli*,* Clostridium disporicum*,* Clostridium novyi*, and *S. baltica*. *Clostridia* is the main bacterial class identified in Budu samples, with percentages of 80.551% and 80.08%. Some of the species in this class are pathogenic. Budu is a food product that has a high salt content, so it can inhibit the growth of pathogens. Meanwhile, fish of poor quality can produce toxins from *Clostridium botulinum* in the product before salting and may be stable in the salted product. A report from Bremner [33] added that the presence of salt in food is an effective way to eliminate toxins because it inhibits pathogenic bacteria such as *Clostridium.* Meanwhile, the discovery of *Staphylococcus,* the second most common genus of bacteria found in fermented fish [24].

This is the first study among many that used metagenomic analysis using 16S rRNA to determine the microbiota biodiversity in Budu, Indonesia. Several shortcomings were noted in the present study. One of the main things is the lack of samples. Taking more samples with a more detailed background (the physicochemical characteristics of the products) could help with a better interpretation of the results. The DNA extraction method used may not be efficient enough to extract all the DNA samples in the Budu, thus the lower number of microorganisms detected. Moreover, the use of 16S rRNA metagenomic analysis was known to detect up to the genus level. The use of shotgun analysis may help to determine the microbiota up to the species level.

## Conclusion

Metagenomic analysis of Budu from different fish, including Pariaman (fresh fish) and Pasaman (frozen fish), showed that the bacterial biodiversity was almost no different. The total number of microbial species in Budu (Pariaman) is higher (227 species) than in Pasaman (153 species). In both buds, LAB from the *Enterococcaceae* family, *Vagococcus* genus, and pathogenic bacteria were found, such as *S. cohnii, P. russeli, C. disporicum,* and* S. baltica. Lentibacillus kimchi, S. cohnii,* and* C. disporicum* were found in both Budu. *Nioella sediminis* and* S. baltica* were found in Budu Pariaman. *Peptostreptococcus russeli* and *C. novyi* were found in Budu Pasaman. The discovery of various species of pathogenic bacteria shows that development is still needed in the Budu production process to improve Budu quality.

## List of abbreviations

ACE, Abundance-based coverage estimators; *C. disporicum, Clostridium disporicum*; *C. novyi, Clostridium novyi*; DNA, deoxyribonucleic acid; LAB, lactic acid bacteria; *L. kimchi, Lentibacillus kimchi*; PCR, polymerase chain reaction; PD, phylogenetic diversity; *N. sediminis, Nioella sediminis*; *P. russeli, Peptostreptococcus russeli*; rRNA, ribosome-ribonucleic acid; *S. cohnii, Staphylococcus cohnii; S. baltica, Shewanella baltica*.
